# Annexin A2: Its Molecular Regulation and Cellular Expression in Cancer Development

**DOI:** 10.1155/2014/308976

**Published:** 2014-01-23

**Authors:** Chi-Yun Wang, Chiou-Feng Lin

**Affiliations:** Institute of Clinical Medicine, College of Medicine, National Cheng Kung University, 1 University Road, Tainan 701, Taiwan

## Abstract

Annexin A2 (ANXA2) orchestrates multiple biologic processes and clinical associations, especially in cancer progression. The structure of ANXA2 affects its cellular localization and function. However, posttranslational modification and protease-mediated N-terminal cleavage also play critical roles in regulating ANXA2. ANXA2 expression levels vary among different types of cancers. With some cancers, ANXA2 can be used for the detection and diagnosis of cancer and for monitoring cancer progression. ANXA2 is also required for drug-resistance. This review discusses the feasibility of ANXA2 which is active in cancer development and can be a therapeutic target in cancer management.

## 1. Introduction

Annexin A2 (also called p36, annexin II, ANXA2, calpactin I, lipocortin II, chromobindin VIII, or placental anticoagulant protein IV), a 36 kDa protein [1], is located on chromosome 15q22.2 [2]. ANXA2 is expressed in some tumor cells, endothelial cells, macrophages, and mononuclear cells. ANXA2 contains three distinct functional regions: the N-terminal region, the C-terminal region, and the core region. The N-terminal region contains the tissue plasminogen activator (tPA)- [3] and S100/A10 (also called p11)-binding site [4]. The core region of ANXA2 contains the calcium and phospholipid-binding site [1, 5]. The C-terminal region contains the F-actin- [6], heparin- [7] and plasminogen-binding sites [8]. The core domain contains four repeats, and each repeat features five alpha-helices. In ANXA2, the core domain possesses two annexin-type calcium-binding sites with the sequence GxGT-[38 residues]-D/E [1, 5]. ANXA2 is activated in a calcium-dependent manner and undergoes a conformational change that exposes a hydrophobic amino acid to form a heterotetramer with p11. This complex shows a high affinity for phospholipids [9]. ANXA2 exists as a monomer or heterotetramer composed of two ANXA2 molecules and two p11 molecules and has four forms, including secrete, membrane-bound, cytoplasmic, and nuclear form. ANXA2 heterotetramer would translocate from the cytoplasm to the extracellular plasma membrane [10]. In general, ANXA2 expressed in the nucleus is cell-cycle-dependent.

## 2. Physiological Function of ANXA2

Intracellular ANXA2 has been reported to play roles in exocytosis [11], endocytosis [12, 13], and membrane trafficking [14]. Knockdown of ANXA2 inhibits cell division and proliferation [15]. Protein kinase C-dependent phosphorylation of ANXA2 blocks the ability of the protein to aggregate phospholipid vesicles but does not affect its lipid vesicle binding properties [16]. Furthermore, ANXA2 is essential for lipid raft formation and signal transduction through its interaction with CD44, a cell receptor for hyaluronic acid that is richly expressed in lipid rafts [17]. Interestingly, recent findings suggest that ANXA2 serves as a ligand for C1q on apoptotic cells [18]. ANXA2 degradation is correlated with cellular apoptosis induced by p53-mediated pathways [19]. As a radioresponsive protein, ANXA2 prevents radiation-induced apoptosis by regulating nuclear factor *κ*B nuclear translocation [20]. Genotoxic stimulations, including gamma radiation, ultraviolet radiation, and etoposide, induce ANXA2 nuclear translocation by reactive oxygen species signaling. This ANXA2 nuclear aggregation prevents genomic damage [21]. ANXA2, as the substrate of thioredoxin, can also act as an endogenous antioxidant. Upon loss of ANXA2, tumor cells undergo apoptosis through proapoptotic p38MAPK (mitogen-activated protein kinase)/c-Jun N-terminal kinase (JNK)/Akt signaling under hydrogen peroxide stimulation [22]. ANXA2 also mediates interferon gamma-induced inflammation via calcium-dependent regulation of protein tyrosine kinase 2 [23]. Knockdown of ANXA2 inhibits interleukin (IL)-6 secretion in a prostate cancer model under starvation stimulation conditions [24]. ANXA2 is important for IL-1*β*- and 12-O-tetradecanoylphorbol 13-acetate-induced tumor necrosis factor-*α* shedding by interacting with ADAM metallopeptidase domain 17. Inhibition of ANXA2 may be a therapeutic strategy for inflammation-associated disease progression [25]. However, ANXA2 may also reduce inflammation through its role as an endogenous inhibitor of phospholipase A2 in mononuclear cells [26].

Extracellular ANXA2 plays a role in phagocytosis [27], fibrinolysis and anticoagulation [28, 29], angiogenesis [29], and cell metastasis [30, 31]. It has been demonstrated that, in homozygous AnxA2-null mice, fibrin accumulation occurs in the microvasculature; however, t-PA-dependent fibrinolytic activity functions well owing to a defect of fibrin and collagen I matrix remodeling and clearance of injury-induced arterial thrombi. Further, under ANXA2 deficient, loss of plasmin leads to failure of activation of metalloprotease-9 and -13 near the migrating cell and may retard cell migration. In addition, bFGF-induced corneal neoangiogenesis and oxygen-induced retinal neoangiogenesis are impaired in the setting of ANXA2 deficiency [29, 32]. These data suggest that ANXA2 is required for angiogenesis and metalloprotease activation, thus promoting cancer cell migration. In macrophages, ANXA2 lowers the surface levels of phosphatidylserine, permitting efficient phagocytosis of apoptotic cells [27]. ANXA2 is also deployed by phagocytes as a receptor for the C1q recognition of apoptotic cells [18]. ANXA2 interacts with plasminogen and tPA, which converts plasminogen to plasmin, thus mediating the lysis of fibrin polymers [28] and extracellular matrix degradation and promotes cell invasion and migration. The antibody blockade of surface ANXA2 can inhibit tumor cell growth and metastasis [31]. However, angiostatin inhibits ANXA2-mediated plasmin generation by competing with plasminogen through the lysine-binding domain of ANXA2 [31].

As for the function of ANXA2 in immune regulation, recent studies show that secreted ANXA2 can activate macrophage for IL-1, IL-6, and TNF-*α* secretion through TLR4/MyD88- and TRIF-dependent pathways [33, 34]. The cytokines from macrophages may promote tumor progression, such as IL-6 for pancreatic cancer [35] and hepatoma [36]. Dendritic cells are the most potent antigen-presenting cells of the immune system. Dendritic cells interact with T and B lymphocytes as well as NK cells to induce and modulate immune responses, including those critically involved in tumor immunosurveillance [37]. Under DAMPs stimuli, such as wear debris from articulating surface, cytosolic ANXA2-S100A10 complex participates in restoring endosomal membrane integrity, thus curtailing NLRP3 inflammasome activation and IL-1 and IL-18 secretion in dendritic cells [38]. It may suggest that, in ANXA2 knockout mice, antigen presenting may be interfered in dendritic cells, thus retarding adaptive immune system activation. IL-18 and IL-1 secretion may be upregulated in ANXA2−/− mice to promote tumor progression and angiogenesis [39, 40].

## 3. Functional Regulation of ANXA2

The membrane association of ANXA2 can be induced by IL-6 in A549 cells via an unknown mechanism [41]. Translocation of ANXA2 to the cell surface can be regulated by serine and tyrosine phosphorylation. For serine phosphorylation, Dubois et al. found that ANXA2 is phosphorylated by Ca^2+^/phospholipid-stimulated kinases such as Ca^2+^-dependent protein kinases C (cPKCs), whereas one of the cPKC substrates, ANXAV, inhibits the phosphorylation of ANXA2 but other cPKC substrates have no effect on kinases that are only activated by phospholipids [42]. On the other hand, tyrosine phosphorylation of ANXA2 is a prerequisite for its translocation to the cell surface [10]. pp60 Src kinase promotes ANXA2 tyrosine phosphorylation in cells treated with platelet-derived growth factor. However, the tyrosine phosphorylation of ANXA2 completely inhibits the ability of the protein to bind to bundle F-actin, suggesting that tyrosine phosphorylation may decrease ANXA2 function, suggesting that the dephosphorylation of ANXA2 might be necessary for protein activation [43]. How is ANXA2 phosphorylation regulated? Recently, Brandherm et al. found that cyclic adenosine monophosphate-induced von Willebrand factor secretion is promoted by serine 11 phosphorylation of ANXA2, which is dephosphorylated via calcineurin-like phosphatase [44]. In addition to phosphorylation, nitration of the ANXA2 tetramer inhibits liposome aggregation [45]. Acetylation of the N-terminal of ANXA2 is required for p11 binding [46]. Abnormal ubiquitination of ANXA2 may promote the metastasis and infiltration of breast cancer cells by inducing high levels of ANXA2 expression [47].

ANXA2 heterotetramerization is required for its extracellular function. N-terminal ANXA2 can stabilize intracellular p11 through direct binding and masking the autonomous p11 polyubiquitination signal, thus preventing proteasomal degradation [48]. The N-terminus of ANXA2 is essential for p11 binding and subsequent heterotetramerization, suggesting an important role for the N-terminus in ANXA2 function [4]. Thus, the regulation of ANXA2 N-terminal cleavage and its role in protein function are important to further our understanding of ANXA2 as a diagnostic or therapeutic tool. The association of ANXA2 with smooth muscle cell membranes can be terminated by proteolytic cleavage. The cleavage is assumed to occur at the N-terminal of ANXA2, and proteolysis is thought to be triggered by the cysteine protease calpain and occurs at nonraft regions of the plasma membrane [49]. Furthermore, the proteolysis of ANXA2 depends on the intracellular concentration of free Ca^2+^ and requires an intact contractile apparatus [49]. In chaperone-mediated autophagy, the cleavage of ANXA2 can be observed in primary human lung fibroblasts [50]. However, the mechanisms of cleavage remain unclear. ANXA2 can be the substrate of plasmin. Thus, plasmin may induce ANXA2 cleavage and dissociation from the ANXA2 heterotetramer. Interestingly, mutating lysine 27 renders ANXA2 resistant to plasmin [51, 52]. *In vitro*, cell cycle inhibition or apoptotic stimuli, such as treatment with chemotherapeutics, ER stress, or growth factor withdrawal, induce ANXA2 cleavage through a synergistic interaction of serine/threonine kinase glycogen synthase kinase-3 and serine protease Omi/HrA2. However, the overexpression of ANXA2 cannot reverse the cleavage [53, 54]. Recently, Yamane et al. [55] found that the asparaginyl endopeptidase legumain cleaves the N-terminus of ANXA2 in the kidney; however, the biological function of cleaved ANXA2 in this context remains unclear [56].

## 4. ANXA2 as a Cancer Biomarker

Increased expression of ANXA2 is frequently observed in a broad spectrum of cancer cells. Overall, ANXA2 is overexpressed in acute lymphoblastic leukemia (ALL) [57], APL [58], breast cancer [59], colorectal carcinoma (CRC) [60], gastric cancer [61], glioma [62], hepatocellular carcinoma (HCC) [63], lung cancer [31, 64, 65], multiple myeloma (MM) [66], oral squamous cell carcinoma (OSCC) [67], and pancreatic cancer [68, 69]. The expression of ANXA2 in relative tissue or cell lines will be discussed below. The upregulation of ANXA2 in cancer may have several clinical applications, including as a diagnostic marker for early detection, a predictive factor for prognosis, or a marker for drug resistance. We summarize the role of ANXA2 in tumors and its clinical relevance in Table 1.

### 4.1. ALL

Actually, compared with the peripheral-blood leukocytes or mononuclear cells from bone marrow from APL patients, the expression of ANXA2 in AML and ALL is lower [58]. On the other hand, MLL-rearranged infant ALL (<1 year of age) is frequently resistant to glucocorticoids. The expression of phosphorylated ANXA2 but not the ANXA2 is obviously higher in prednisolone-resistant ALL patient samples and cell lines (SEM and KOPN8) than in sensitive patients and cells (RS4; 11 and BEL-1). Moreover, the inhibition of ANXA2 phosphorylation by silencing Src family proteins Fyn and Lck sensitized cells to drug prednisolone. Thus, the inhibition of ANXA2 phosphorylation may be a therapeutic target for the treatment of glucocorticoid resistance in this highly aggressive type of leukemia [57].

### 4.2. APL

Compared to other leukemic cell types, leukemic cells from patients with APL or NB4 and HL60 cell lines display elevated levels of ANXA2 protein expression, as detected by a fluorescent-tagged antibody. The t(15; 17)-positive APL cells stimulated the generation of cell-surface, t-PA-dependent plasmin twice as efficiently as the t(15; 17)-negative cells. An abnormally high level of ANXA2 on APL cells increases the production of plasmin, a fibrinolytic protein. Thus, overexpression of ANXA2 may contribute to the hemorrhagic complications of APL [58]. However, plasmin-mediated hyperfibrinolysis may also be corrected by all-trans retinoic acid (ATRA) or ATRA plus arsenic trioxide therapy in patients with APL [70] by downregulating ANXA2 expression [71].

### 4.3. Breast Cancer

ANXA2 is undetectable in normal and hyperplastic ductal tissue samples. By contrast, ANXA2 is consistently expressed in invasive breast cancer and ductal carcinoma *in situ*. Furthermore, ANXA2 is selectively expressed in the invasive/metastatic MDA-MB231 cell but not in the noninvasive/nonmetastatic MCF-7. ANXA2-dependent, localized plasmin generation by human breast cancer cells could contribute to angiogenesis and metastasis [59, 72]. In addition, Zhang [73] has shown that the knockdown of ANXA2 in MDA-MB-435s cells downregulates the levels of plasmin, S100A10, and c-Myc and inhibits breast cancer proliferation and invasion [55]. A recent study indicated that ubiquitination could cause ANXA2 overexpression in breast cancer tissue compared to normal tissue [47]. Furthermore, the overexpression of phosphoANXA2 in Herceptin resistant and Her-2 negative breast cancer tissue contributes to Herceptin-resistance [74].

### 4.4. CRC

In 105 cases of primary colorectal carcinoma tissues, ANXA2 is overexpressed in the cancer cell membrane of the carcinoma cells more than in tumor stroma fibrous tissue, the muscularis propria, the vessel wall, and the adjacent normal bowel wall. Moreover, the expression of ANXA2 is positively correlated with histological type, tumor size, depth of invasion, and pathological tumor-node-metastasis (pTNM) stage [60]. In 150 pairs of colorectal carcinoma tissue and the corresponding adjacent normal tissue, ANXA2 is mainly located in the plasma membrane, and dramatically upregulated in tumor cells compared with normal tissue [75]. In addition to these clinical data, ANXA2 is highly expressed in human CRC cell lines including SW480, SW620, HCT116 and HT29, compared with the normal colonic epithelial cell line NCM460 [75]. However, the expression of ANXA2 in cell lines, HT29-P, HT29-LM, KM12-C and KM12-SM, is not apparently correlated with metastatic potential [60]. In addition, butyrate-induced cell differentiation and growth arrest in the human colon adenocarcinoma cell lines BCS-TC2, BCS-TC2.2, and HT-29 is correlated with decreased ANXA2 expression, although no decrease was observed in Caco-2 cells [76]. Recently, ANXA2 is identified as a novel receptor for gastrin and progastrin (PG) peptides, which mediate the growth stimulatory effects of autocrine and exogenous gastrin and PG peptides on intestinal epithelial and colon cancer cells [77]. The expression of ANXA2 is strong in poorly differentiated tumor tissues and is significantly correlated with disease recurrence and patient survival. Thus, ANXA2 may act as a biomarker with both diagnostic and prognostic potential for CRC patients [75].

### 4.5. Gastric Cancer

There is a clinical association between upregulated ANXA2 and tumorigenesis in gastric carcinoma. However, there is little understanding of the molecular function of ANXA2 in this context. Emoto et al. demonstrated that thirty-three percent of 153 primary gastric carcinoma patients are immunopositive for ANXA2; however, overexpression of ANXA2 is correlated with differentiation, lymph node metastasis, and venous invasion [61]. It has also been reported that, in 133 of 436 gastric cancer cases, the ANXA2 expression is elevated in gastric cancer tissues compared with noncancerous tissues. ANXA2 is overexpressed strongly in the cell membrane of primary cancer cells and weakly in the cytoplasm of carcinoma cells [78]. ANXA2 and c-erbB (erythroblastic leukemia viral [v-erb-b] oncogene homolog)-2 overexpression are significantly correlated, and patients with ANXA2 had poor prognoses. Multivariate analysis showed that coimmunopositivity of ANXA2 and c-erbB-2 independently indicates a poor prognosis [61, 78]. However, the functional significance of ANXA2 overexpression in gastric cancer has not been investigated.

### 4.6. Glioma

ANXA2 protein is highly expressed in human high grade, but not in low grade, primary gliomas tissues. The expression of ANXA2 is highest in glioblastoma multiforme, intermediate in anaplastic astrocytomas, and lowest in astrocytomas. In contrast to the usual cytoplasmic localization of ANXA2, distinct nuclear staining has been found in many of the specimens [62]. Knockdown of ANXA2 on GL261 mouse glioma cell impairs tumor progression *in vivo*, but lack of ANXA2 in stromal cells has no effect on glioma growth [79]. The migration of U87MG and U373MG cells, but not proliferation and adhesion, is significantly inhibited following ANXA2 depletion by small interfering RNA (siRNA) [79, 80]. Furthermore, as a consequence of its interaction with S100A10, ANXA2 can serve as a binding protein for procathepsin B on the surface of breast tumor and glioma cells, which may further facilitate tumor invasion and metastasis [30].

### 4.7. HCC

ANXA2 is rarely detected in either normal or chronic hepatic tissues but is overexpressed transcriptionally and translationally in tumorous and nontumorous regions of HCC (primarily localized in cancer cells), especially in poorly differentiated HCC (*P* < 0.01). Interestingly, both the tyrosine phosphorylation and protein expression of ANXA2 are elevated in HCC compared to normal or cirrhosis tissue [63]. Furthermore, the levels of HAb18G/CD147, an immunoglobulin family member enriched on the surface of tumor cells, are reported to be correlated with the invasion, metastasis, growth, and survival of malignant cells. ANXA2 can interact with HAb18G/CD147 to regulate matrix metalloprotease (MMP)-2 expression and migration and influence the invasive potential and cytoskeletal rearrangement of human HCC cells [81]. ANXA2 is significantly increased in the sera of HCC compared with the sera from healthy, benign tumor, hepatitis, and cirrhosis controls and other malignant tumors. Notably, increased concentrations of ANXA2 are observed in 83.2% (79/95) of early stage (median, 24.32 ng/*μ*L) and 78.4% (58/74) of alpha-fetoprotein (AFP)-negative (median, 24.09 ng/*μ*L) patients. Thus, ANXA2 may act as an independent serological candidate for the early detection of HCC, especially in early stage cases with normal serum AFP [64, 82]. At present, in addition to detecting the serum expression of ANXA2, the addition of sinusoidal ANXA2 expression to the already established marker panel (glypican 3, heat shock protein 70, and glutamine synthetase) increases the reliability and objectivity of HCC diagnosis in liver biopsies [83].

### 4.8. Lung Cancer

ANXA2 is located on the long arm of chromosome 15 (15q21) [2]; 15q is one of the most frequently acquired chromosome arms in nonsmall cell lung carcinoma [84]. ANXA2 is upregulated in the doxorubicin-selected multidrug resistant small cell lung cancer cell line H69AR [85] compared with parental cells. Intense ANXA2 immunoreactivity is detected in lung adenocarcinoma and squamous cell carcinoma, compared to noncancerous control. In one study, the presence of ANXA2 autoantibody in sera can be detected in lung cancer patients with squamous cell carcinoma (33%) and lung adenocarcinoma (60%) [41]. The same study found that the generation of anti-ANXA2 antibodies is correlated with the serum levels of IL-6 in lung cancer patients. In nonsmall cell lung cancer (NSCLC), A549-derived tumors, ANXA2 is highly expressed near proliferative cells but not in either the necrotic region or in apoptotic cells [54]. Knockdown of ANXA2 results in the blockage of proliferation and migration in A549 cells [86]. ANXA2 is highly expressed in NSCLC and is positively correlated with poor prognosis [65]. The tumor size, pathological grade, pT status, pN status, and pleural invasion are positively correlated with ANXA2, most likely through the role of ANXA2 in the regulation of MMP-2 and cathepsin B expression [64, 86]. Thus, ANXA2 may be a prognostic marker and a therapeutic target for NSCLC.

### 4.9. MM

Myeloma cells from MM patients display an increased expression of ANXA2 compared to plasma cells from normal subjects. In addition, ANXA2 expression in the MM cell lines U266 and RPMI8226 is elevated compared to other hematologic cell lines. Furthermore, ANXA2 has been shown to be required for the antiapoptosis, proliferation, and invasive potential of the MM cell lines U266 and RPMI8226 [66]. A recent study found that the expression of ANXA2 is higher in myelomatous (6.67 ± 6.47 ng/mL) than in normal BM (1.4 ± 1.43 ng/mL) serum [87]. Thus, ANXA2 levels, which are easily measured in serum, could be a clinical prognostic factor for multiple myeloma.

### 4.10. Pancreatic Cancer

Vishwanatha et al. found that in normal pancreas, ANXA2 expression is observed in ductal and ductular cells and there is no expression in acinar or islet cells. ANXA2 mRNA and protein levels in five established human pancreatic adenocarcinoma cell lines are elevated 5- to 15-fold compared with normal pancreas. However, higher expression of ANXA2 (2- to 8-fold) is observed in three primary pancreatic tumors and one metastatic tumor compared with normal pancreas [69]. Furthermore, Diaz et al. have used coimmunoprecipitation and plasmin activity assays to demonstrate that ANXA2 specifically binds to t-PA on the extracellular membrane of pancreatic cancer cells (PANC-1 and SK-PC-1 cells), where it activates local plasmin production and tumor cell invasion [88]. The presence of anti-ANXA2 antibodies in patient serum is positively correlated with disease-free survival greater than 36 months. Furthermore, in cancer tissue, the surface expression of ANXA2 is associated with pancreatic cancer development [89]. Moreover, a positive relationship between the cellular levels of S100A6 and the membrane localization of ANXA2 provides a possible mechanism for ANXA2-related increases in pancreatic cancer cell motility [90]. Detection of the anti-ANXA2 antibody in patient serum may be a good prognostic indicator.

### 4.11. Other ANXA2-Overexpressed Cancer Cells

ANXA2 is overexpressed in human B-cell lymphoma cell lines but is significantly reduced in Raji, OMA-BL-1 and REH cells compare with normal B-cells [91]. Ohno et al. have shown that ANXA2 is upregulated in primary clear cell renal carcinoma cells (ccRCCs) compared with normal renal cortex; 47.4% of primary ccRCCs tissue and 87.5% of metastatic tumors are positive for ANXA2. The ANXA2 expression in primary tumors is significantly associated with a later stage and a higher nuclear grade. Finally, in patients without metastasis, the 5-year metastasis-free rate in patients with ANX2-positive tumors is significantly lower than in those with ANX2-negative tumors [92]. In oral squamous cell carcinoma (OSCC), there is an elevated ANXA2 mRNA and protein expression in cancerous HB56 and HB96 cells over human immortalized oral epithelial cells. Overexpression of ANXA2 in tumor tissue over adjacent noncancerous epithelial cells is positively correlated with tumor size and tumor recurrence, but negatively correlated with tumor differentiation grades [67, 93]. However, detection of ANXA2 only in OSCC cannot be an independent prognostic marker for disease-specific survival [67].

## 5. ANXA2 Acts as a Tumor Suppressor Gene in Some Cancer Types

ANXA2 expression is decreased in esophageal squamous cell carcinoma (ESCC) [94, 95], osteosarcoma [96], and prostate cancer [97].

### 5.1. ESCC

The mRNA and protein expression of ANXA2 is lower in only 22 ESCC tumor cells in the patient tissues than that in its paired normal counterpart [95], and this downregulation is significantly correlated with lymphoid node metastasis (*P* < 0.05) and pathological differentiation (*P* < 0.05) [94]. The aberrant expression of ANXA2 in differentiation and metastasis may be evaluated as a potential target for clinical biomarkers after a large, properly controlled clinical study.

### 5.2. Osteosarcoma

In osteosarcoma, the ANXA2 mRNA and protein levels are inversely correlated with metastatic potential in a subset of human osteosarcoma tumor specimens. However, overexpression of ANXA2 in SaOSLM2 cells did not alter several *in vitro* phenotypes that are often used to assess metastatic potential, including motility, adhesion, and proliferation. This study demonstrated, surprisingly, that ANXA2 is involved in the differentiation-induced mineralization process of osteoblastic cells *in vitro* and may decrease osteosarcoma aggressiveness [96].

### 5.3. Prostate Cancer

ANXA2 heavy (p36) and light (p11) chains in 31 of 31 prostate cancer specimens are undetectable by immunohistochemistry, suggesting a loss of ANX2A expression in cancer cells, whereas basal cells are positively stained [98]. ANXA2 expression is also reduced or lost in prostate cancer cell lines, such as LNCAP, PC3, and DU145, and so forth, compared with primary normal human prostate; however, overexpression of ANXA2 in DU145 or LNCaP inhibits cell migration [97]. However, the aberrant accumulation of nuclear ANXA2 through the mutation of its nuclear export signal retards the proliferation of LNCaP cells [99]. Recently, ANXA2/ANXA2 receptor axis has been shown to promote the adhesion of prostate cancer to osteoblasts and endothelial cells [100]. In a clinical study, the ANXA2 expression is significantly lower in prostate cancer compared to benign prostatic hyperplasia (*P* < 0.01). However, in some high-grade tumors, ANXA2 facilitates the adhesion-dependent or -independent growth of prostate cancer through the MAPK pathway [100] or interleukin-6 (IL-6) secretion [24]. Currently, lower ANXA2 expression in prostate cancer patients, compared to that in benign prostatic hyperplasia, correlates with an advanced clinical stage, more frequent recurrence, and regional lymph node and distant metastasis and may serve as a useful biomarker [73].

## 6. ANXA2 and Tumor Behavior

Some of the earliest studies of ANXA2 demonstrated its expression on the APL surface and its role as a receptor for tPA and plasminogen following fibrinolysis [1]. In pancreatic cancer, ANXA2 is highly expressed, acts as a tPA receptor to activate plasminogen, and consequently increases the invasiveness of pancreatic cancer cells by altering the extracellular matrix [101]. Similarly, ANXA2 is highly expressed in the breast cancer cell line MDA-MB231 (highly invasive) compared with MCF-7 (poorly invasive) [59]. Furthermore, strong cell surface expression of ANXA2 is observed in tumor cells and stromal cells surrounding the blood vessels. The ANXA2 partner S100A10 has also been found to play a significant role in the invasiveness of fibrosarcoma cells [102].

In addition to metastasis and invasion, previous studies have shown that ANXA2 is involved in tumor cell proliferation [55, 66, 103]. The effect of ANXA2 on the proliferation of different cancer cells is summarized in Table 2. Vishwanatha et al. showed that the increased expression of ANXA2 is limited to proliferating ductular adenocarcinoma and that ANXA2 expression is colocalized with cells that express PCNA [69]. ANXA2 silencing inhibits cell division and proliferation following the blockade of DNA synthesis in the cervical cancer cell line HeLa as well as the multiple myeloma cell lines U266 and RPMI8226 [15, 66]. Nuclear ANXA2 acts as a primer recognition protein and is involved in DNA replication [104–106]. siRNA targeting by ANXA2 reduces the levels of p11 and c-Myc and causes cell cycle arrest in MDA-MB-435 cells [55]. Furthermore, ANXA2 mediates the effects of gastrin/PG peptides that stimulate the growth of autocrine and exogenous gastrin and PG peptides on intestinal epithelial and colon cancer cells [77]. ANXA2 is required for the growth of prostate cancer and acts through the MAPK pathway [100] or by upregulating IL-6 secretion [24]. However, the overexpression of ANXA2-green fluorescent protein in DU145 prostate cancer cells does not affect cell-cycle progression, as assessed by BrdU incorporation assays [97]. Nuclear export signal mutation causes the aberrant accumulation of nuclear ANXA2 and retards the proliferation of LNCaP cells [99]. We have further demonstrated a novel role for ANXA2 in NSCLC cell proliferation by facilitating the cell cycle through the maintenance of JNK/c-Jun-inhibited p53 [65].

## 7. Conclusion

ANXA2 has been studied for many years, especially in tumors. Recently, the potential role of ANXA2 in immunology gives a hint on the interaction between immune cells and cancer cells in the microenvironment. In this paper, we summarize the expression and structure of ANXA2 as well as its function in cancer cells or primary cancer tissues. In the future, ANXA2 may be a potential prognostic factor, a potential diagnostic factor for cancer, and a therapeutic target for new drug development after analysis in large scale, well-controlled, prospective clinical trials.

## Figures and Tables

**Table 1:** Summary of the role of ANXA2 acting as a marker in tumors.

Cancer	Expression	Method	Clinical relevance	Reference
ALL	Up	WB, qRT-PCR	Resistant to prednisolone	[57]
APL	Up	IFA	Hemorrhagic complications	[58]
B-cell lymphoma	Up	WB	None	[91]
ccRCC	Up	IHC	Metastasis	[92]
Breast	Up	Northern blot, WB	Migration and invasion Herceptin-resistance	[59, 74]
Breast	Up	IHC	Metastasis Neoangiogenesis	[59, 72]
Cervical	Up	IHC	Poor survival rate Poor differentiation Chemoresistance	[107]
CRC	Up	IHC	Poor survival rate Recurrence Poor differentiation	[75]
ESCC	Down	IHC, qRT-PCR, WB	Poor differentiation Lymph node metastasis	[94]
Gastric cancer	Up	IHC	Depth of invasion Lymph node metastasis and distant metastasis TNM stage	[61, 78]
Glioma	Up	WB	Invasion and angiogenesis	[69]
HCC	Up	ELISA	Diagnostic marker in early stage	[82]
HCC	Up	IHC, WB	Poor differentiation Diagnostic marker	[63]
Lung	Up	IHC, IFA	Tumor size and grade Pleural invasion Poor prognosis	[64, 86]
MM	Up	Flow cytometry	Poor prognosis Poor survival	[87]
Osteosarcoma	Down	RT-PCR, WB, IFA	Poor differentiation Metastasis	[96]
OSCC	Up	IHC, WB, qRT-PCR	Tumor size and frequent recurrence, well differentiation	[67]
Pancreatic	Up	ELISA	Good survival	[89]
Pancreatic	Up	Northern blot, WB	Proliferation	[69]
Prostate	Down	IHC, WB	Advanced clinical stage More frequent recurrence Regional lymph node and distant metastasis	[73, 100]

WB: western blot; qRT-PCR: quantitative reverse transcription polymerase chain reaction; IFA: immunofluorescence assay; IHC: immunohistochemistry; ELISA: enzyme-linked immunosorbent assay.

**Table 2:** Summary of the effects of ANXA2 on cancer cells.

Cancer	Description	Reference
Breast cancer	The siRNA targeting ANXA2 of MDA-MB-435s cells downregulates the levels of plasmin, S100A10, and c-Myc and thus retards breast cancer proliferation and invasion	[55]
Colorectal carcinoma	ANXA2 downregulation is not due to proteasome activation and seems to be related to the butyrate-induced cell proliferation arrest	[76]
Colorectal carcinoma	ANXA2 can mediate growth stimulatory effects of autocrine and exogenous gastrin and PG peptides on intestinal epithelial and colon cancer cells	[77]
NSCLC	Knockdown of ANXA2 upregulates p53 and causes cell cycle arrest so efficiently as to retard cell proliferation	[86]
Pancreatic cancer	Increased expression of ANXA2 colocalizes with cells that express PCNA	[69]
Prostate cancer	Aberrant accumulation of nuclear ANXA2 retards proliferation of LNCaP cells	[99]
Prostate cancer	ANXA2 may facilitate the growth of prostate cancer *in vitro* and *in vivo* by the MAPK pathway	[100]
Prostate cancer	Intracellular ANXA2 contributes to secretion of IL-6 in prostate cancer, and this effect requires the C-terminus of ANXA2	[24]
